# Editorial: Reviews in pediatric urology 2024

**DOI:** 10.3389/fped.2025.1743431

**Published:** 2025-11-26

**Authors:** Sujit Kumar Chowdhary

**Affiliations:** Indraprastha Apollo Hospitals, New Delhi, India

**Keywords:** pediatric, urology, review, patient-centered care, meta-analysis

Pediatric urology has seen a rapid transformation in the last 2 decades, largely due to the convergence of biological insights and technological innovation, with an aim to provide healthcare access across divergent socioeconomic landscapes. The *Reviews in Pediatric Urology 2024* Research Topic captures this dynamic evolution through 14 articles. Spanning genetic mechanisms, novel therapies, and systemic challenges, these works showcase the advances that redefine care for children with urological conditions worldwide.

The Research Topic begins with a deep dive into biological mechanisms. Oliveira et al.'s study uses animal models to unravel the genetic, developmental, and hormonal drivers of variations in sexual characteristics, highlighting conserved pathways across species. This foundational work informs clinical approaches to congenital anomalies, resonating with Gozar et al.'s systematic review of urethral multiplicity, which catalogs 250 cases to guide precise diagnostics and tailored surgical interventions. Similarly, Mao et al. challenge assumptions about non-palpable testes, showing that blind-ended vas deferens does not always indicate testicular absence, urging thorough exploration to address malignancy risks. Jeries et al.'s review of the pediatric urobiome reveals microbial influences on urinary health and disease, with implications for conditions like urinary tract infections. These studies collectively underscore the need for biological precision to inform diagnosis and treatment, bridging bench science to bedside care.

Therapeutic advancements form a second theme, addressing both functional and structural urological challenges. Cheng et al.'s meta-analysis of parasacral transcutaneous electrical nerve stimulation (PTENS) for overactive bladder (OAB) demonstrates a 1.90-fold higher response rate than conventional treatments, with reduced constipation, offering a non-invasive alternative. Frainey et al. highlight onabotulinumtoxinA (BTX-A) as a transformative tool for pediatric neurogenic lower urinary tract dysfunction (NLUTD), supported by its 2021 FDA approval for neurogenic detrusor overactivity. Haddad et al.'s review of alpha-adrenergic antagonists in pediatric nephrolithiasis reports faster stone expulsion and reduced pain, while Paloian et al.'s study on X-linked hypophosphatemic rickets (XLH) notes burosumab's role in managing nephrocalcinosis risks. On the surgical front, Pokharkar et al.'s 10-year study of robotic surgery for abdominal tumors achieves a 90% survival rate, showcasing precision and reduced recovery times. These innovations highlight a spectrum of interventions, from minimally invasive to cutting-edge surgical techniques, tailored to diverse pediatric needs.

Innovation alone cannot ensure impact; it must be supported by systems that promote equity and competence. This collection also identifies systemic barriers and educational needs. Blumenthal et al. advocate for ethical, sustainable short-term surgical outreach, emphasizing capacity building in low-resource settings to ensure equitable access. Davidson et al. explore animal and simulation models for fetal and neonatal surgery training, balancing ethical concerns with the need for realistic practice environments. Teehan et al.'s creation of a community advisory board for pediatric bladder health addresses school-based barriers to lower urinary tract symptoms (LUTS) management, promoting community-driven interventions. Bhatia et al.'s identification of multidisciplinary stakeholders for hypospadias care underscores the need for holistic, lifelong follow-up, while Okanlami et al.'s qualitative study reveals toileting challenges for college students with physical disabilities, emphasizing self-advocacy and accessible facilities. These articles collectively call for systemic improvements, from training to infrastructure, to enhance patient-centered care.

The clinical implications of these studies are profound, yet, limitations persist. Small sample sizes (e.g., Pokharkar et al.'s 20 patients, Paloian et al.'s 13), methodological heterogeneity, and reliance on specific cohorts (e.g., North American or Asian populations) raise generalizability concerns. Translational gaps also remain, with computational findings needing functional validation. Future research must address these gaps through larger, multicenter trials and standardized protocols to validate therepeutic innovations and modifications to enhance consistency.

